# Essential Features of Responsible Governance of Agricultural Biotechnology

**DOI:** 10.1371/journal.pbio.1002453

**Published:** 2016-05-04

**Authors:** Sarah Hartley, Frøydis Gillund, Lilian van Hove, Fern Wickson

**Affiliations:** 1 School of Sociology and Social Policy, University of Nottingham, Nottingham, United Kingdom; 2 GenØk Centre for Biosafety, Tromsø, Norway; City University London, UNITED KINGDOM

## Abstract

Agricultural biotechnology continues to generate considerable controversy. We argue that to address this controversy, serious changes to governance are needed. The new wave of genomic tools and products (e.g., CRISPR, gene drives, RNAi, synthetic biology, and genetically modified [GM] insects and fish), provide a particularly useful opportunity to reflect on and revise agricultural biotechnology governance. In response, we present five essential features to advance more socially responsible forms of governance. In presenting these, we hope to stimulate further debate and action towards improved forms of governance, particularly as these new genomic tools and products continue to emerge.

## Abstract 2

Agricultural biotechnology continues to generate controversy, and new approaches to governance are needed for the next generation of techniques and applications. This Perspective proposes five essential features of responsible governance.

*This Perspective is part of the Public Engagement in Science Series*.

## An Opportunity to Reflect on Agricultural Biotechnology Governance

Agricultural biotechnology continues to generate significant controversy. Much of this controversy goes beyond questions about human and environmental safety to include concerns regarding intellectual property and monopoly ownership rights, consolidating corporate control over seed markets and the food chain, consumers’ and farmers’ right to know and choose, and challenges concerning the coexistence of different agricultural production systems. Existing regulatory frameworks struggle to address this wide range of concerns, as they largely rely on scientific risk assessment of human and environmental health only.

The new wave of genomic tools and products place additional pressure on existing governance frameworks and provide a particularly useful opportunity to reflect on and revise agricultural biotechnology governance. Some scientists argue that new genomic tools, such as gene editing and the CRISPR/Cas9 method, will revolutionize biotechnology by allowing easy, cheap, precise, and predictable genetic modification, while critics remain concerned about social and ethical implications and how existing regulatory frameworks will apply [1,2]. Alongside the development of new tools, the next wave of agricultural biotechnology products (beyond genetically modified [GM] crops) are now being approved for open field trials and human consumption. For example, in 2014, the United States approved the first agricultural GM insect, a GM diamondback moth, for open release. Applications have also been made for releases of agricultural GM insects in Europe and Australia.

Given the entrenched controversy that has persisted around the use of GM crops, serious reflection and changes to governance are needed for new biotechnology tools and products in agricultural settings. Discussion about the regulatory status of the new suite of developments is underway, highlighting the uncertainty that exists about whether these new genomic tools and products fall outside existing definitions of GM organisms (GMOs) and regulatory requirements [3]. However, decisions about whether these products and techniques fall inside or outside existing regulatory frameworks are unlikely to address or appease the persistent controversy.

In response to the limitations of current approaches to governance, calls have been made for the full range of actors, including diverse public stakeholders, to carefully consider the risks as well as the social and ethical concerns associated with the next wave of agricultural biotechnologies [2]. Alongside international responses to human genome editing [4], some examination of governance of these new genomics tools in agricultural biotechnology is also taking place, with a focus on GM insects [5], gene drive research, and genome editing in nonhuman organisms [6]. However, these efforts are narrowly focused on traditional regulatory oversight, bioethics, and risk mitigation strategies and exclude the full range of potentially interested actors.

Drawing on extensive scholarship on technology governance and the agricultural biotechnology controversy from across the fields of science and technology studies, responsible research and innovation, and procedural ethics, we identify five common and essential features that are interdependent rather than mutually exclusive and needed in order to advance more socially responsible forms of governance. These are: commitment to candour, recognition of underlying values and assumptions, involvement of a broad range of knowledge and actors, consideration of a range of alternatives, and preparedness to respond. In presenting and elaborating on these features below, we hope to stimulate further debate and action towards improved forms of governance, particularly as new genomic tools and products continue to emerge.

## Five Essential Features of Responsible Governance of Agricultural Biotechnology

### 1. Commitment to Candour

Responsible governance of agricultural biotechnologies first and foremost requires honesty and humility about several factors embroiled in the controversy. These factors include: the scope and quality of the available scientific knowledge, the underlying motivations for a technology’s use and development, the realisability of claimed benefits, the range of concerns at stake (e.g., including those beyond physical risks), the information available in application dossiers, and potential conflicts of interests in assessment and decision making.

The lack of candour in current risk regulatory frameworks is a foundation for public concern [7,8]. Public stakeholders are not necessarily risk averse in the face of uncertainty. However, experience of past technological safety failures has sensitised people to the limits of scientific knowledge and made them sceptical of those advocating complete knowledge and an ability to predict and control technological risks in complex socioecological systems. A lack of truthfulness concerning the limits of scientific knowledge, motivations, expected benefits, and the basis of conflict can lead to significant misunderstandings and mistrust between scientists, policy makers, and the public. Certainty and predictability are typically considered to be a measurement of competence [9]; however, candidly recognising and truthfully representing scientific uncertainties and the full range of concerns at stake does not reflect a lack of competence. Rather, such candour allows debate to move beyond the unhelpful illusion of technological control and open up for a broader and more inclusive discussion about the role of technology in addressing socioecological challenges [7].

Social science research has suggested several ways to encourage honesty and humility in the governance practices of agricultural biotechnologies. For example, work by Andy Stirling has usefully articulated different types of scientific uncertainty and identified available methods for understanding, identifying, and addressing them [10]. Furthermore, some of the factors making biotechnology particularly prone to inflated hype have been highlighted (e.g., pressures to publish, the increasing commercialisation agenda, and media representations), and concrete changes to address these have also been identified (such as altered incentive structures, independent resources, enhanced scepticism, and a robust and critical role for media) [11].

### 2. Recognition of Underlying Values and Assumptions

Responsible governance also requires that actors reflect upon how values and assumptions shape both science and innovation and risk assessment and management. Recognising underlying values and assumptions in innovation and governance systems can make explicit the diverse ways there are to approach how desirable futures are imagined, how problems and solutions are framed and understood, and how risk-based science and assessment are performed.

Risk assessment, as the foundation of existing governance frameworks for agricultural biotechnology, is regularly claimed to be “science-based” and free of values. Without recognition, the values-based choices made by risk assessors and the scientific experts advising them are hidden from public scrutiny but continue to generate controversy [8,12]. Recognising the significance of values-choices and the underlying assumptions of different actors would reveal divergent views and allow them to be negotiated and addressed directly rather than hiding them within a narrow debate about human and environmental risk. This would in turn enable more transparent decision making and effective dialogue between innovators, risk assessors, risk managers, policy makers, and publics.

One approach advocated by social scientists to help facilitate the recognition of underlying values and assumptions is to create meeting places where actors with different knowledge and ideas are invited to contribute to shaping agricultural biotechnology decisions in both scientific research and risk regulation. This is important because it is often in meetings between people of different views, backgrounds, and understandings that underlying values and assumptions are revealed. One illustrative example is the approach taken in Socio-Technical Integration Research, in which scholars from the social sciences and humanities are embedded into laboratories to help scientists reflect upon their research decisions, recognise their value choices, explore alternatives, and integrate broader social and ethical considerations into their routine activities [13].

### 3. Involvement of a Broad Range of Knowledges and Actors

A commitment to candour and acknowledgement of values and assumptions facilitates decision making to be opened up to a plurality of perspectives, views, and diverse types of knowledge from a broad range of actors, including both different scientific disciplines (e.g., molecular biology and ecology) and different stakeholders (e.g., farmers, citizens, and civil society organisations).

When the debate about agricultural biotechnology is confined to a technical assessment of risks to human and environmental health, it limits who can legitimately participate in decision-making processes, privileging technical experts [12,14]. The inclusion of nontechnical experts is currently confined to the end of the risk assessment process, when public stakeholders are invited to comment on expert-defined assessments of environmental and human health risk without much potential for influence [12]. However, as we have argued, the debate about agricultural biotechnology is not only a technical debate about physical risks: it involves other ethical and social concerns [14]. Opening up innovation governance to a broader range of knowledge sources and perspectives allows social and ethical concerns to be directly incorporated and addressed in decision making. However, it also has normative value by making the process more democratically legitimate and can substantively improve decision making by creating a more comprehensive knowledge base [15,16].

The importance of engaging the public in the governance of agricultural biotechnology has received increased attention and experimentation over the years, not only from social scientists but also from policy makers and nongovernmental organisations. This has led to work with diverse methods such as citizen juries, consensus conferences, science cafes, and public dialogues and meetings. While many of these attempts at public engagement have received significant criticism for their narrow framing, selection of participants, limited impact, and/or their search for legitimation rather than genuine debate [7,12,17], there is arguably a learning process in motion. For example, calls have been made to move engagement efforts further “upstream” in the innovation process to ensure that these discussions do not occur too late (e.g., when trajectories have been set, precious research funds invested, and decisions effectively made) [18]. Examples such as the Rothamsted Research institute meeting with stakeholders before running experimental GM wheat field trials arguably represents a small step in the right direction [19].

### 4. Consideration of a Range of Alternatives

Responsible governance of agricultural biotechnology should not just candidly and inclusively consider the risks and societal and ethical concerns associated with a particular technology but should also consider the range of alternative ways to formulate and frame the problems at stake as well as the range of alternative approaches to solving them.

Agricultural systems are under severe stress from converging problems associated with soil deterioration, water scarcity, chemical pollution, climate change, and population growth. At present, policies to address these problems typically focus on the problems in isolation and call almost exclusively on science and technology narrowly defined for solutions [20]. Assessing individual technologies in isolation for only their potential risks encourages a narrow focus on issues of risk governance rather than a broader interest in innovation governance or “problem governance” and does not support the development of “wicked solutions” [21] capable of addressing multiple concerns simultaneously.

Social scientists again have relevant work to offer in the practical implementation of this feature, such as the development of methods designed to facilitate the consideration of a range of technological alternatives and policy options. One promising method here is Multicriteria Mapping, which uses web-based software to help scientists, risk assessors, or others to explore different options available for addressing a defined problem on the basis of a range of flexible evaluative criteria [22]. This technique not only enables a clear picture to be developed about how different stakeholders view the available alternatives and how they weight different evaluative criteria, it also captures how they see the uncertainties involved and how these uncertainties affect their preferences and decision making. This allows biotechnologies to be compared with a range of other technologies for their potential to address the problem at hand against diverse evaluative criteria [23].

### 5. Preparedness to Respond

For all of the above features to function effectively, innovators, risk researchers, regulators, and policy makers need to be willing and prepared to consider and respond to societal needs and concerns as well as to new scientific knowledge, changing values, diverse interpretations of potential consequences, and shifting socioecological conditions.

This preparedness to respond to matters of societal concern and changing conditions is important not only for ensuring the democratic accountability of science and technology in liberal democracies but also as a means to enhance reversibility, adaptability, and resilience in innovation and policy systems in the face of change [16]. The inherent limitations of scientific knowledge and our inability to fully predict and control agricultural biotechnologies in dynamic natural systems places further emphasis on the need for preparedness and a willingness to respond.

Responding to societal concerns and changing socioecological conditions requires moving beyond dichotomous stop-go decisions towards more nuanced considerations of how, when, under what conditions, and in what directions, we may wish to move forward. For example, in a recent case in the US, societal concerns prompted researchers at Cornell University to reexamine the timetable for releasing the GM diamondback moth in open field trials. Despite receiving a regulatory permit for open releases, researchers delayed open field trials, framing the decision as “responsible science” [24]. This means that the response to societal concern was not simply about moving forward with the technology in the face of concern or stopping it entirely but also about considering questions regarding the appropriate speed of development and what further research may be required and conducted so as to address the concerns raised.

## Conclusion

Agricultural biotechnology, like all innovation, is a future-oriented endeavour, and therefore its governance necessarily requires balancing the desire to harness potential future benefits with sensitivity to existing uncertainties and a constructive engagement with diverse societal needs and concerns. The five essential features of responsible governance that we provide here are intended to help stimulate a broader discussion about how to imagine and enact this balancing through developing scientifically and socially responsible governance of agricultural biotechnologies. Such discussion is particularly salient as new genomic tools and products emerge into a milieu where the controversies that embroil GM crops remain unresolved. Adopting these essential features of agricultural biotechnology governance is no small task. We have provided some illustrative examples from social science research of attempts to move governance practices of agricultural biotechnologies in this direction. While we recognise that each of these practical examples have their limitations and possible critiques, we nevertheless see them as small steps towards the type of deep cultural change that is needed to fully enact the features we advocate. Although these essential features are not intended to be comprehensive or definitive, they are interconnected, and we would argue that they need to be consistently cultivated, encouraged, and reinforced, in both individuals and institutions over time, until innovation and governance systems gradually evolve into more responsible forms that successfully serve a broader range of socioecological goals.
